# Multifactorial analysis of risk factors for foot ulcers in patients with neurovascular complications of diabetes

**DOI:** 10.3389/fendo.2024.1399924

**Published:** 2024-10-11

**Authors:** Zibo Fan, Jinyan Huang, Yue Liu, Hao Xie, Qinfeng Yang, Yue Liang, Hong Ding

**Affiliations:** ^1^ Department of Nursing, Nanfang Hospital, Southern Medical University, Guangzhou, Guangdong, China; ^2^ School of Nursing, Southern Medical University, Guangzhou, Guangdong, China; ^3^ Department of Anesthesiology, Nanfang Hospital, Southern Medical University, Guangdong Provincial Key Laboratory of Precision Anaesthesia and Perioperative Organ Protection, Guangzhou, Guangdong, China; ^4^ Division of Orthopaedic Surgery, Department of Orthopaedics, Nanfang Hospital, Southern Medical University, Guangzhou, Guangdong, China; ^5^ Department of Nursing, Nanping First Hospital affiliated with Fujian Medical University, Nanping, Fujian, China

**Keywords:** neurovascular disease, diabetes, diabetic foot ulcer, risk factors, health management

## Abstract

**Background:**

Diabetic foot ulcers (DFU) are a major complication associated with significant morbidity and mortality. While numerous studies have investigated risk factors for these ulcers in general, few have focused specifically on patients with Neurovascular Complications of Diabetes. This study aimed to evaluate the prevalence and risk factors for DFU in this specific population.

**Methods:**

We analyzed data from the National Institutes of Health (NIS) database for the years 2017-2019, involving a cohort of 161,834 patients aged over 18 who were diagnosed with neurovascular complications of diabetes. Demographic characteristics (age, gender, ethnicity), hospital characteristics, comorbidities, and other relevant data were included for analysis. A binary logistic regression model was generated to identify independent risk factors for DFU.

**Results:**

The prevalence of DFU among patients with neurovascular complications of diabetes was 29.4% during the period from 2017 to 2019. Compared to patients without DFU, those with DFU had longer hospitalization times and higher costs. The multiple regression analysis revealed that Iron-deficiency anemia (OR, 1.10; 95% CI, 1.01-1.11; *P*=0.019), Hypertension (OR, 1.07; 95% CI, 1.03-1.11; *P*=0.001), Obesity (OR, 1.08; 95% CI, 1.06-1.11; *P*<0.001), Peripheral vascular disorders (PVD) (OR, 1.69; 95% CI, 1.65-1.74; *P*<0.001), Osteomyelitis (OR, 7.10; 95% CI, 6.89-7.31; *P*<0.001), Tinea pedis (OR, 1.89; 95% CI, 1.59-2.26; *P*<0.001), Sepsis (OR, 1.24; 95% CI, 1.20-1.28; *P*<0.001), and onychomycosis (OR, 1.26; 95% CI, 1.13-1.42; *P*<0.001) were independent predictors for DFU in this population.

**Conclusion:**

The study found a high prevalence of DFU in patients with neurovascular complications of diabetes. Identifying and addressing risk factors such as deficiency anemia, hypertension, obesity, PVD, infections, and foot conditions may contribute to reducing the prevalence of DFU in this vulnerable population.

## 1 Introduction

Diabetes mellitus (DM) is a pervasive global health challenge, with 463 million cases diagnosed globally (1). It’s characterized by multi-organ complications that expedite functional decline and mortality (2). Recent research highlights neurodegenerative and neurovascular complications as key areas of interest. Microvascular disease, a common consequence of diabetes, encompasses a range of pathological changes and symptoms, traditionally categorized as “microvascular complications.” However, there’s a shift towards classifying these as “neurovascular complications,” emphasizing the intricate interplay between microvascular and neural factors (3). In patients with diabetes, PAD in the lower extremities, often accompanied by neurological issues, is a primary contributor to DFU and tissue damage. Notably, 90% of diabetic foot ulcers are attributed to neurogenic ischemia (4). Furthermore, neuropathy impairs microvascular reactivity, exacerbating ulceration, healing impairments, and infection vulnerability (5, 6).

In pre-diabetes, microvascular dysfunction can be detected prior to macrovascular or occlusive arterial disease (6). As diabetes progresses, it disrupts the structure and function of small blood vessels, triggering microvascular diseases that ultimately cause ischemic skin tissue damage and various pathological changes and symptoms (7). The sympathetic nervous system plays a crucial role in regulating skin microcirculation during posture changes, impacting arteriovenous anastomosis and precapillary function (8). Dysfunction in endothelium-dependent microvascular regulation and sympathetic denervation-induced autonomic neuropathy, linked to sweating impairments, may contribute to diabetic foot complications. Given that dry, cracked skin is prone to infections and ulcerations, safeguarding microcirculatory function is paramount (6).

DFU, as one of the most destructive complications of DM, has been associated with numerous negative consequences, including significant impairments in quality of life, decreased mobility and independence, increased incidence and mortality rates, and a substantial burden on healthcare resources (9). Therefore, accurately identifying risk factors and implementing effective preventive measures for DFU is crucial. However, research on risk factors for DFU in patients with diabetes has yielded inconsistent results, with few studies specifically investigating these factors in patients with neurovascular complications of diabetes. To address this gap, we analyzed clinical data of patients with neurovascular disease of diabetes from the NIS database between 2017 and 2019.

By analyzing the clinical characteristics of hospitalized DFU patients with neurovascular lesions, this study aims to investigate thoroughly the potential risk factors for DFU and provide more targeted support for prevention and treatment. The findings are expected to contribute to the scientific basis for improving the rehabilitation of patients with diabetes and reducing the incidence of DFU.

## 2 Methods

### 2.1 Data source and characteristics

**Figure 1:**
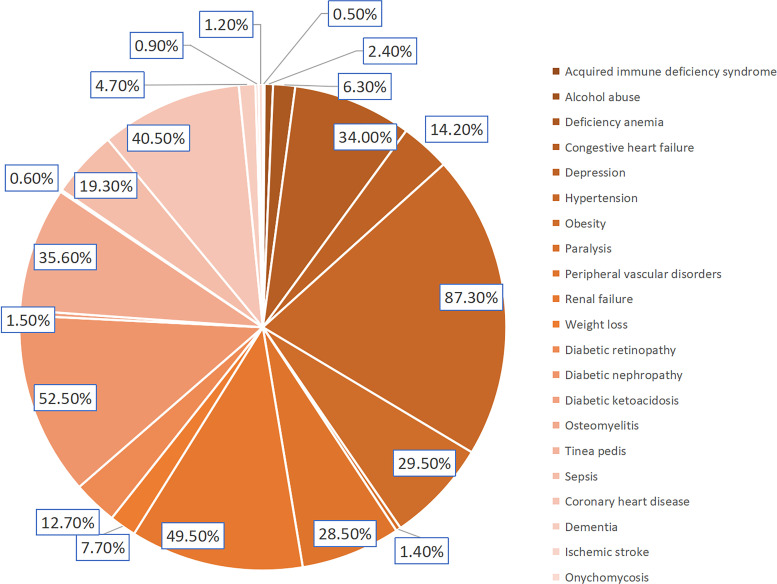
Incidence of DFU related comorbidities.

The data for this study was sourced from the NIS database, a 20% random sample of all hospitalized patients in the United States. This large, nationally representative sample makes the NIS ideal for conducting descriptive research, obtaining national estimates, analyzing healthcare costs, studying rare diseases, and understanding trends over time. We extracted essential patient information, including age, gender, and race, as well as basic hospital information and some related comorbidities from the database (**Table 1**, **Figure 1**).

**Table 1:** Variables used in binary logistic regression analysis.

Variables Categories	Specific Variables
**Patient demographics**	Age (≤64 years and ≥65 years), sex (male and female), race (White, Black, Hispanic, Asian or Pacific Islander, Native American and Other), type of diabetes (type 2 diabetes)
**Hospital characteristics**	Type of admission (non-elective, elective), bed size of hospital (small, medium, large), teaching status of hospital (nonteaching, teaching), location of hospital (rural, urban), type of insurance (Medicare, Medicaid, private insurance, self-pay, no charge, other), location of the hospital (northeast, Midwest or north central, south, west)
**Comorbidities**	AIDS, alcohol abuse, deficiency anemia, hypertension, obesity, peripheral vascular disorders, weight loss, osteomyelitis, tinea pedis, sepsis, onychomycosis, smoke

AIDS, Acquired immunodeficiency syndrome.

### 2.2 Population

**Figure 2:**
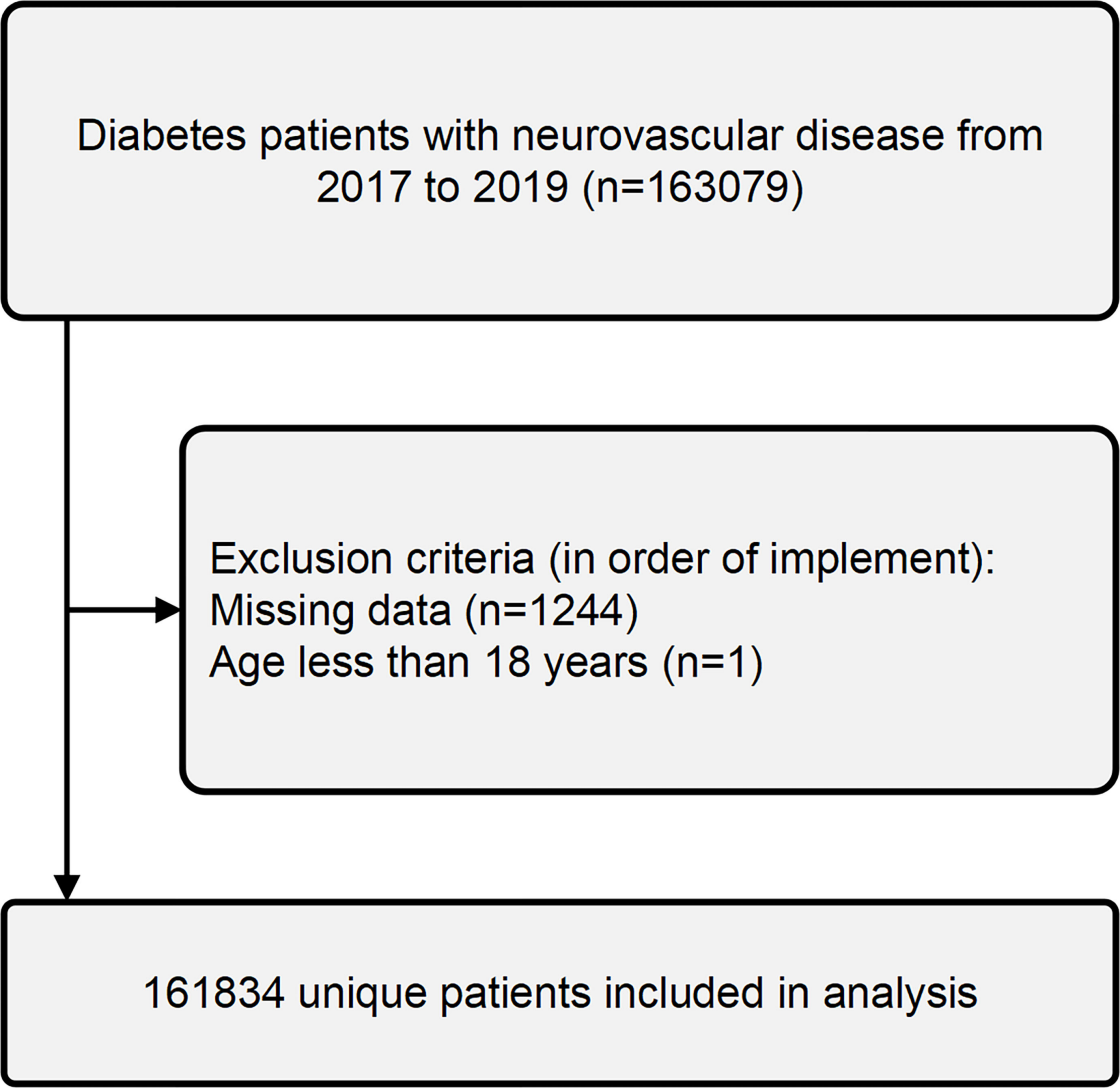
Procedures for patient inclusion and exclusion.

The data was collected from the NIS database for patients hospitalized between January 1, 2017, and December 31, 2019, resulting in a dataset of 163,079 individuals. After excluding individuals with missing data (n=1,245), the final study population comprised 161,834 patients with neurovascular complications of diabetes (**Figure 2**). Inclusion criteria included patients diagnosed with both diabetes mellitus and neurovascular disease based on the 10th revision of the International Statistical Classification of Diseases (ICD-10) codes. We excluded patients under 18 years old and those with non-pressure chronic ulcers of the thigh, calf, other lower leg regions, or unspecified lower leg locations. We compared demographic and clinical characteristics of patients with and without DFU to identify potential risk factors for developing DFU.

### 2.3 Statistical analysis

Statistical analyses were performed using IBM SPSS 26.0 software (IBM Corp., 2019). The study population was divided into two groups: DFU (n=47,604; 29.4%) and non-DFU (n=114,230; 70.6%). Categorical variables were compared using chi-square tests, while continuous variables were analyzed with unpaired Student’s t-tests. Descriptive analysis was conducted using frequency, constituent ratio, mean, and standard deviation. Measurement data conforming to a normal distribution are presented as mean ± standard deviation (SD), while measurement data that do not conform to a normal distribution are reported as median. The count data were expressed as percentage. Logistic regression models were used to identify factors associated with DFU. Univariate analysis was first performed to identify significant variables, followed by multivariate analysis using variables with p-values < 0.05 from the univariate analysis as covariates. Statistical significance was set at *P*< 0.05.

## 3 Results

Among the 161,834 patients analyzed, 37.6% were male (n=60,818) and 62.4% were female (n=101,016). The mean age was 63.15 years in the DFU group and 67.11 years in the non-DFU group (**Table 2**). The DFU group had a significantly higher proportion of males (70.3%) compared to the non-DFU group (**Table 2**).

**Table 2:** Characteristics and results of diabetes patients with neurovascular diseases (2017-2019).

Characteristics	DFU		No DFU	P
Total (n=count)	47604		114230	
Total incidence (%)	29.4			
Age (median, years)	63 (55,72)		67 (59, 76)	<0.001
Age group (%)				
18-44	6.50		3.80	<0.001
45-64	48.20		36.70	
65-74	27.20		30.50	
≥75	18.10		29.00	
Gender (%)				
Male	70.30		59.10	<0.001
Female	29.70		40.90	
Race (%)				
White	60.90		63.90	<0.001
Black	17.10		17.60	
Hispanic	15.00		11.90	
Asian or Pacific Islander	1.50		1.70	
Native American	1.20		0.90	
Other	4.30		4.00	
Number of Comorbidity (%)				
0	-		-	<0.001
1	1.60		0.70	
2	8.20		4.40	
≥3	90.20		95.00	
Characteristics	DFU		No DFU	P
Type of insure (%)				
Medicare	62.80		74.80	<0.001
Type of insure (%)				
Medicaid	14.50		10.10	
Private insurance	17.30		11.70	
Self-pay	3.20		1.50	
No charge	0.30		0.10	
Other	1.80		1.70	
Bed size of hospital (%)				
Small	20.10		19.10	<0.001
Medium	30.60		29.80	
Large	49.30		51.10	
Elective admission (%)	88.20		86.40	<0.001
Type of hospital (%)				
teaching	73.60		73.80	0.262
non teaching	26.40		26.20	
Location of hospital (%)				
urban	93.50		93.40	0.561
rural	6.50		6.60	
Region of hospital (%)				
Northeast		17.30	16.10	<0.001
Midwest or North Central		25.60	27.40	<0.001
South		37.60	37.20	
West		19.50	19.20	
Type of diabetes (type 2 diabetes)		94.40	94.30	0.397
Smoke (%)		39.40	43.50	<0.001

Based on the demographic analysis, we performed multiple regression analysis on relevant variables. The results showed that age ≥ 65 years (OR: 0.67, 95% CI: 0.67-0.71, *P*< 0.001), female gender (OR: 0.66, 95% CI: 0.65-0.67, *P*< 0.001), and smoking (OR: 0.77, 95% CI: 0.76-0.79, *P*< 0.001) were significantly associated with DFU (**Table 3**).

**Table 3:** Related risk factors of diabetes patients with neurovascular disease and diabetic foot ulcers.

Variable	Multivariate Logistic Regression		
	OR	95% CI	P
Age ≥65 years old	0.67	0.67-0.71	<0.001
Female	0.66	0.65-0.67	<0.001
Race			
White	0.93	0.90-0.95	<0.001
Black	1.11	1.04-1.11	<0.001
Hispanic	0.80	0.73-0.88	<0.001
Asian or Pacific Islander	1.17	1.05-1.30	0.004
Native American	1.02	0.96-1.08	0.568
Other	Ref	——	——
Variable	Multivariate Logistic Regression		
	OR	95% CI	P
Number of Comorbidity			
1	0.88	0.78-0.98	0.025
2	0.71	0.64-0.80	<0.001
Number of Comorbidity			
≥3	Ref	——	——
Type of insurance			
Medicare	1.25	1.20-1.30	<0.001
Medicaid	1.24	1.20-1.28	<0.001
Private insurance	1.53	1.42-1.65	<0.001
Self-pay	1.51	1.18-1.94	0.001
No charge	1.01	0.93-1.10	0.779
Other	Ref	——	——
Bed size of hospital			
Small	0.97	0.94-1.00	0.048
Medium	0.91	0.88-0.94	<0.001
Large	Ref	——	——
Elective admission	0.74	0.71-0.76	<0.001
Teaching hospital	0.97	0.95-1.00	0.054
Urban hospital	0.99	0.94-1.04	0.577
Region of hospital			
Northeast	1.00	0.97-1.04	0.998
Midwest or North Central	0.90	0.87-0.93	<0.001
South	0.91	0.88-0.95	<0.001
West	Ref	——	——
Smoke	0.77	0.76-0.79	<0.001

OR, Odds ratio; CI, Confidence interval.

Univariate analysis identified statistically significant associations (*P*< 0.05) between DFU and all the investigated comorbidities and complications, suggesting a correlation between DFU and these factors. Multiple regression analysis revealed several independent risk factors for DFU, including Iron-deficiency anemia (OR: 1.10, 95% CI: 1.01-1.11, *P*= 0.019), hypertension (OR: 1.07, 95% CI: 1.03-1.11, *P*= 0.001), obesity (OR: 1.08, 95% CI: 1.06-1.11, *P*< 0.001), PVD (OR: 1.69, 95% CI: 1.65-1.74, *P*< 0.001), osteomyelitis (OR: 7.10, 95% CI: 6.89-7.31, *P*< 0.001), tinea pedis (OR: 1.89, 95% CI: 1.59-2.26, *P*< 0.001), sepsis (OR: 1.24, 95% CI: 1.20-1.28, *P*< 0.001), and onychomycosis (OR: 1.26, 95% CI: 1.13-1.42, *P*< 0.001).

An analysis of patient outcomes revealed that, among patients with neurovascular complications of diabetes, the length of hospitalization and total cost were significantly higher in the DFU group compared to the non-DFU group (**Table 4**).

**Table 4:** Prognosis and results of patients with neurovascular diseases and diabetes (2017-2019).

Characteristics	DFU	No DFU	P
**LOS (median, d)**	7.00 (4.00, 11.00)	5.00 (3.00, 8.00)	<0.001
**TOTCHG (median, $)**	63749.50 (34876.25, 115224.50)	48023.50 (26183.50, 91938.00)	<0.001
**Died (%)**	1.40	2.70	<0.001

LOS, Length of stay; TOTCHE, Total charge.

## 4 Discussion

**Figure 3:**
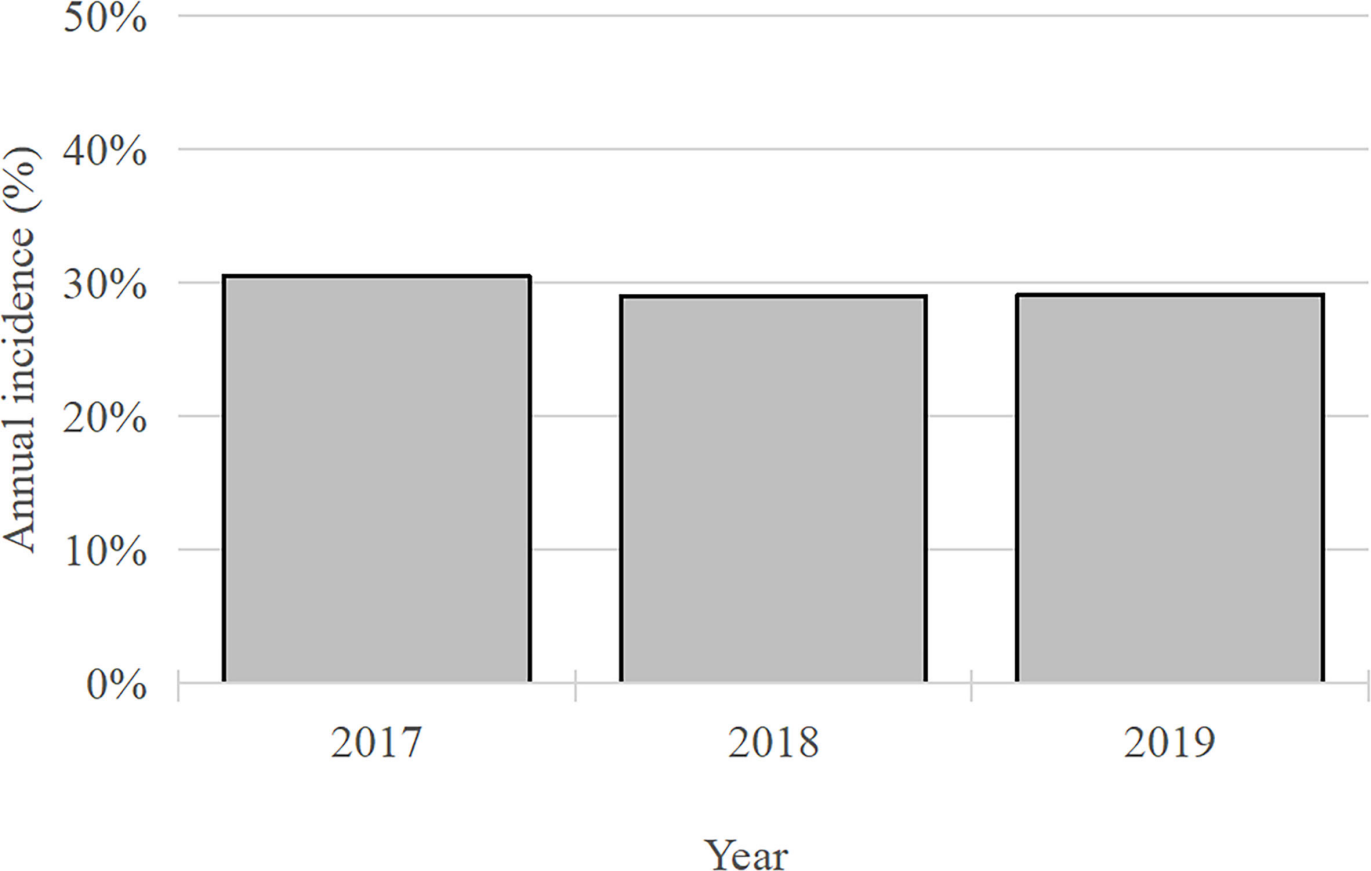
Incidence rate of foot ulcers in diabetes patients with neurovascular disease and diabetes.

This study found a 29.4% prevalence of DFU among patients with neurovascular complications of diabetes between 2017 and 2019 (**Figure 3**). This prevalence is lower than reported rates in Ethiopia (31.1%) and Nigeria (41.1%) (10, 11). These disparities may stem from differences in sample size, geographic location, and sociocultural factors among study populations. Additionally, our study focused specifically on patients with neurovascular complications of diabetes, a population underrepresented in previous research. Multiple regression analysis identified Iron-deficiency anemia, hypertension, obesity, PVD, osteomyelitis, tinea pedis, sepsis, and onychomycosis as independent risk factors for DFU in this study (**Table 5**).

**Table 5:** Relationship between diabetic foot ulcers and comorbidities.

Comorbidities	Univariate Analysis			Multivariate Logistic Regression		
	No DFU	DFU	P	OR	95% CI	P
Acquired immune deficiency syndrome	469 (0.4%)	221 (0.5%)	0.131	1.00	0.84-1.18	0.959
Deficiency anemia	7651 (6.7%)	2986 (6.3%)	0.002	1.10	1.01-1.11	0.019
Hypertension	100688 (88.1%)	41547 (87.3%)	<0.001	1.07	1.03-1.11	0.001
Obesity	33729 (29.5%)	14025 (29.5%)	0.792	1.08	1.06-1.11	<0.001
Peripheral vascular disorders	23502 (20.6%)	13564 (28.5%)	<0.001	1.69	1.65-1.74	<0.001
**Comorbidities**	**Univariate Analysis**			**Multivariate Logistic Regression**		
	**No DFU**	**DFU**	**P**	**OR**	**95% CI**	**P**
Weight loss	9737 (8.5%)	3675 (7.7%)	<0.001	1.04	1.00-1.08	0.075
Osteomyelitis	7622 (6.7%)	16944 (35.6%)	<0.001	7.10	6.89-7.31	<0.001
Tinea pedis	311 (0.3%)	269 (0.6%)	<0.001	1.89	1.59-2.26	<0.001
Sepsis	16438 (14.4%)	9197 (19.3%)	<0.001	1.24	1.20-1.28	<0.001
Onychomycosis	973 (0.9%)	551 (1.2%)	<0.001	1.26	1.13-1.42	<0.001

OR, Odds ratio; C, Confidence interval.

We investigated potential risk factors for DFU in the study population. The observed male predominance aligns with findings from earlier studies, such as the work by Edgar et al. (12). This may suggest a higher susceptibility to foot trauma among males, potentially contributing to a greater prevalence of DFU in this population (13). Moreover, the mean age in our study was higher compared to that reported by Hokkam et al. (14). This difference could be attributed to factors, including increased life expectancy in Europe and the United States. Besides, the prevalence of DFU among patients residing in rural areas was slightly lower than that observed in urban areas, which contradicts previous findings reported by Chowdhury et al. (11), which might be due to the relatively small sample sizes in both urban and rural groups, potentially affecting the accuracy of DFU prevalence estimates in these subgroups.

Our study revealed that patients with neurovascular complications of diabetes and Iron-deficiency anemia have a 1.10-fold greater risk of DFU compared to patients with diabetes without anemia, echoing previous research on anemia’s link to heightened foot complications (15–18). Anemia in diabetics exacerbates DFU risk via mechanisms like tissue hypoxia, metabolic dysfunction, and reduced glucose uptake, leading to hyperglycemia. Chronic hyperglycemia causes vascular damage, potentially resulting in foot complications and even mortality (19). Charcot arthropathy, a severe diabetic-specific arthropathic condition, arises from neurological and orthopedic factors, causing neuropathy and bone/joint deformities. Diabetic peripheral neuropathy drives foot complications, especially Charcot arthropathy, emphasizing its pivotal role. A study by Madeline Lyons. et al. found anemia increases the risk of Charcot arthropathy in diabetics by 1.798-fold, with complication risks escalating as the deformity progresses (20). However, the precise mechanisms underlying anemia’s impact on Charcot arthropathy are unclear, necessitating further research to unravel this complex relationship and develop targeted therapies for diabetics.

Our study found a higher prevalence of DFU among patients with neurovascular complications of diabetes and hypertension. This association aligns with previous research demonstrating the link between hypertension and both microvascular and macrovascular complications in diabetes (21). Hypertension, or high blood pressure, can damage the blood vessels, including those in the feet, leading to reduced blood flow and impaired perfusion. This can result in ischemia, a condition where the tissues do not receive enough oxygen and nutrients due to inadequate blood supply. Ischemia is a crucial factor in the development and worsening of DFU, as it impairs wound healing and increases the risk of infection (22). Additionally, the combined effects of hypertension and hyperglycemia can lead to inadequate blood supply and decreased local oxygen saturation in the lower limbs, ultimately weakening tissue resistance and immunity.

Our study further found that patients with neurovascular complications of diabetes and obesity experience a 1.08-fold increased risk of developing DFU compared to non-obese patients. These findings are consistent with previous research conducted in Ethiopia, Kenya, and Malaysia, highlighting regional concordance in this association (23). Obesity is associated with several metabolic derangements that can contribute to the development of DFU. Firstly, obesity often leads to insulin resistance, a key feature of type 2 diabetes (24). Secondly, obesity can exacerbate inflammation, which can impair wound healing and promote the development of infections. Additionally, excess weight can put pressure on the feet, leading to increased shear forces and the formation of DFU, particularly in patients with neuropathy (10).

Moreover, patients with PVD were found to have a 1.69-fold higher risk of DFU compared to those without PVD. PVD encompasses arterial and venous system diseases, and its complex nature often leads to asymptomatic DFU in the early stages, progressing to chronic non-healing ulcers with prominent tissue loss in later stages. PVD, such as atherosclerosis and peripheral artery disease (PAD), can cause narrowing or blockage of blood vessels in the legs and feet. This results in reduced blood flow and ischemia, which can impair wound healing and increase the risk of infection. The impact of PAD, which involves the narrowing or blockage of arteries in the lower limbs leading to reduced blood flow (25), is well-documented. It has been reported that PAD contributes to 50-70% of DFU cases and is a significant risk factor for delayed wound healing, infection, amputation, and mortality in both type 1 and type 2 diabetes (26).

Furthermore, our study revealed a 7.01-fold increased risk of DFU in patients with osteomyelitis compared to those without. This association is supported by evidence demonstrating a strong link between diabetes and the increased risk of both acute and chronic osteomyelitis (27). Osteomyelitis is a bone infection that can occur in the feet of patients with diabetes. This infection can destroy bone tissue, causing structural instability and increasing the risk of ulceration. Osteomyelitis can also impair wound healing and promote the development of sepsis, a life-threatening condition characterized by widespread infection in the body (28). Several studies support the bidirectional relationship between DFU and osteomyelitis. Lavery et al. (29) found that the presence of osteomyelitis negatively impacts diabetic foot infection outcomes, potentially promoting DFU development. Similarly, Zhang et al. (30) identified diabetes foot osteomyelitis (DFO) as a complex complication arising after DFU and requiring surgical intervention, further establishing its role as an independent risk factor for DFU recurrence. Furthermore, Yesil et al. (28) emphasize the significance of osteomyelitis as a risk factor for major amputation among DFU patients, extending its impact beyond DFU occurrence.

Our study findings also suggest that both onychomycosis and tinea pedis are independent risk factors for DFU. Onychomycosis can affect the feet of patients with diabetes. This infection can cause thickening and distortion of the nails, which can lead to pressure points and ulceration. Additionally, onychomycosis can disrupt the normal anatomy of the foot, making it more susceptible to injury and infection (31). Tinea pedis can disrupt the skin’s barrier function, making it more susceptible to injury and infection. In patients with diabetes, tinea pedis can lead to the development of DFU, particularly in areas of the feet that are already compromised by neuropathy or ischemia (32). This aligns with research by Akkus et al., who reported a significantly higher prevalence of fungal infections between the toes, soles, and toenails in patients with DFU compared to those without (31). They further highlight that poor blood glucose control and PVD in patients with diabetes increase susceptibility to fungal infections, potentially contributing to DFU development.

Sepsis poses a severe complication in vulnerable DFU patients, elevating the risk of non-traumatic amputation, multi-organ failure, and even death. Sepsis is characterized by a systemic inflammatory response that can lead to organ dysfunction and, in some cases, death. Sepsis can exacerbate the underlying neurovascular complications of diabetes, further impairing wound healing and increasing the risk of amputation (33).

While this study did not find an association between diabetes type and DFU, research by Mariam et al. suggests that diabetes type is a strong predictor of DFU. Their findings indicate a 2.58-fold increased risk of developing DFU in patients with type 2 diabetes compared to type 1 (34). Additionally, studies conducted in Nigeria, Egypt, and Asia have reported a significant association between type 2 diabetes and DFU occurrence (10, 35, 36). These discrepancies might stem from differences in the utilized databases and study populations.

## 5 Implications and limitations

The strengths of this study lie in its substantial sample size and utilization of the NIS database, which enables precise identification of patients with neurovascular complications of diabetes. However, it is important to acknowledge several limitations, including retrospective biases and data constraints. To address these, rigorous data cleaning, statistical adjustments, and discussions on generalizability were employed. Nevertheless, the ICD coding system’s evolution and inherent limitations pose challenges, affecting diagnosis specificity and ulcer severity scores’ accuracy. This may lead to undercoding of complications and misleading conclusions. Moreover, the underutilization of low-cost diagnostic measures further compromises assessments. To mitigate these issues, validation using multiple data sources is essential. Additionally, the HCUP-NIS database’s lack of clinical fine-grained data and focus on hospitalization data hinder in-depth analysis and risk adjustment, impacting medical quality and outcome assessments. Consequently, comprehensive analysis of the medical system is limited, posing challenges to fully understanding patient diagnosis and treatment. Our findings have significant clinical and public health implications for patients with neurovascular complications of diabetes. By identifying DFU risk factors, we can inform targeted screening and prioritize high-risk patients for foot exams and education. Modifiable risk factors can guide preventive interventions and lifestyle modifications. Furthermore, novel biomarkers may pave the way for personalized medicine. A multidisciplinary approach, focusing on both neurological and vascular components, is crucial for comprehensive care, emphasizing collaboration among healthcare providers. Ultimately, our findings underscore the importance of early DFU detection and management to reduce morbidity, disability, and healthcare costs. Prevention efforts and timely interventions can improve patient outcomes and alleviate financial burdens.

## 6 Conclusion

This study found a high prevalence of DFU among patients with neurovascular complications of diabetes between 2017 and 2019. Several factors, including iron-deficiency anemia, hypertension, obesity, PVD, osteomyelitis, tinea pedis, sepsis, and onychomycosis, were associated with DFU. Our findings on DFU risk factors align with prior studies, emphasizing the crucial role of glycemic control. While age, gender, and comorbidities may exert varying impacts due to study differences, our work underscores the significance of neuropathy-focused paradigm. We advocate a holistic approach that integrates neural and vascular factors in DFU prevention and treatment. Future research should strive for large-scale, standardized studies to clarify DFU risks and identify universal predictors. To this point in the field of health care, to ensure that patients with accessibility and improve the diagnosis, treatment and the result is very important. Given Singh AV et al. Research, future research should give priority to meet strict regulatory standards of safety and efficacy evaluation (37). Our research contributes to refining DFU guidelines and evidence-based medicine, aiming to alleviate the global DFU burden and elevate patient quality of life. Moreover, our findings deepen the understanding of DFU risks and challenge existing paradigms. This knowledge can empower preventative strategies and ultimately reduce the prevalence of DFU.

## Data Availability

The raw data supporting the conclusions of this article will be made available by the authors, without undue reservation.

## References

[1] Wu B Niu Z Hu F. Study on risk factors of peripheral neuropathy in type 2 diabetes mellitus and establishment of prediction model. Diabetes Metab J. (2021) 45:526–38. doi: 10.4093/dmj.2020.0100 PMC8369209 34352988

[2] Gasecka A Siwik D Gajewska M Jaguszewski MJ Mazurek T Filipiak KJ. Early biomarkers of neurodegenerative and neurovascular disorders in diabetes. J Clin Med. (2020) 9:2807. doi: 10.3390/jcm9092807 32872672 PMC7564566

[3] Araszkiewicz A Zozulinska-Ziolkiewicz D. Retinal neurodegeneration in the course of diabetes-pathogenesis and clinical perspective. Curr Neuropharmacology. (2016) 14:805–9. doi: 10.2174/1570159x14666160225154536 PMC5333590 26915422

[4] Mariadoss AVA Sivakumar AS Lee CH Kim SJ. Diabetes mellitus and diabetic foot ulcer: Etiology, biochemical and molecular based treatment strategies via gene and nanotherapy. BioMed Pharmacother. (2022) 151:113134. doi: 10.1016/j.biopha.2022.113134 35617802

[5] Barwick AL Tessier JW Janse de Jonge X Ivers JR Chuter VH. Peripheral sensory neuropathy is associated with altered postocclusive reactive hyperemia in the diabetic foot. BMJ Open Diabetes Res Care. (2016) 4:e000235. doi: 10.1136/bmjdrc-2016-000235 PMC4947724 27486520

[6] Balasubramanian G Vas P Chockalingam N Naemi R. A synoptic overview of neurovascular interactions in the foot. Front Endocrinol (Lausanne). (2020) 11:308IF: 5.2 Q1. doi: 10.3389/fendo.2020.00308IF:5.2Q1 32528410 PMC7256167

[7] Edmonds M Lázaro-Martínez JL Alfayate-García JM Martini J Petit JM Rayman G. (2018). 10.1016/S2213-8587(17)30438-2 29275068

[8] Stirban A. Microvascular dysfunction in the context of diabetic neuropathy. Curr Diabetes Rep. (2014) 14:541. 10.1007/s11892-014-0541-x 25189434

[9] Alexiadou K Doupis J. Management of diabetic foot ulcers. Diabetes Ther. (2012) 3:4. doi: 10.1007/s13300-012-0004-9IF:3.8Q2 22529027 PMC3508111

[10] Ogbera AO Adedokun A Fasanmade OA Ohwovoriole AE Ajani M. The foot at risk in Nigerians with diabetes mellitus-the Nigerian scenario. Int J Endocrinol Metab. (2005) 4:165–73.

[11] Edgar J Peters G Lavery LA Armstrong DG Diabetic lowerextremity infection. Influence of physical, psychological, and social factors. J Diabetes Complications. (2005) 19:107–12. 10.1016/j.jdiacomp.2004.06.002 15745841

[12] Norris FH. Epidemiology of trauma: frequency and impactof different potentially traumatic events on different demographic groups. J Consult. Clin Psychol. (1992) 60:409–18. 10.1037//0022-006x.60.3.409 1619095

[13] Hokkam EN. Assessment of risk factors in diabetic foot ulceration and their impact on the outcome of the disease. Prim Care Diabetes. (2009) 3:219–24. doi: 10.1016/j.pcd.2009.08.009 19783493

[14] Chowdhury HK Khan MH Wadud JR. Risk factors for thedevelopment of diabetic foot ulcer in Bangladesh. Diabetes Res Clin Pract. (2000) 50:282.

[15] Boyko EJ Ahroni JH Stensel V Forsberg RC Davignon DR Smith DG. A prospective study of risk factors for diabetic foot ulcer. The seattle diabetic foot study. Diabetes Care. (1999) 22:1036–42. 10.2337/diacare.22.7.1036 10388963

[16] El-Shazly M Zaki A Nicolucci A. Care-related risk factors for chronic diabetic complications in developing countries: a case from Egypt. Public Health. (2002) 116:289–96. 10.1038/sj.ph.1900855 12209405

[17] Bresäter LE Welin L Romanus B. Foot pathology and risk factors for diabetic foot disease in elderly men. Diabetes Res Clin Pract. (1996) 32:103–9. 10.1016/0168-8227(96)01201-6 8803488

[18] Xiong J Hu H Guo R Wang H Jiang H. Mesenchymal stem cell exosomes as a new strategy for the treatment of diabetes complications. Front Endocrinol (Lausanne). (2021) 12:646233. doi: 10.3389/fendo.2021.646233 33995278 PMC8117220

[19] Wright JA Oddy MJ Richards T. Presence and characterisation of anaemia in diabetic foot ulceration. Anemia. (2014) 2014:104214. doi: 10.1155/2014/104214IF:2.9 25197565 PMC4134799

[20] Lyons M McGregor PC Pinzur MS Adams W Wilkos-Prostran L. Risk reduction and perioperative complications in patients with diabetes and multiple medical comorbidities undergoing charcot foot reconstruction. Foot ankle Int. (2021) 42:902–9. doi: 10.1177/1071100721995422 33629589

[21] American Diabetes Association. Cardiovascular disease and risk management: standards of medical care in diabetes-2020. Diabetes Care. (2020) 43:S111–s134. doi: 10.2337/dc20-S010 31862753

[22] Sowers JR Epstein M Frohlich ED. Diabetes, hypertension, and cardiovascular disease: an update. Hypertension (Dallas Tex: 1979). (2001) 37:1053–9. 10.1161/01.hyp.37.4.1053 11304502

[23] Nyamu PN Otieno CF Amayo EO McLigeyo SO. Risk factors and prevalence of diabetic foot ulcers at Kenyatta National Hospital, Nairobi. East Afr Med J. (2003) 80:36–43. 12755240 10.4314/eamj.v80i1.8664

[24] Amogne W Reja A Amare A. Diabetic foot disease in Ethiopian patients: a hospital based study. Ethiopian J Health Dev. (2011) 25:17–21.

[25] Conte MS Bradbury AW Kolh P White JV Dick F Fitridge R. Global vascular guidelines on the management of chronic limb-threatening ischemia. J Vasc Surg. (2019) 69:3S–125S.e40. 31159978 10.1016/j.jvs.2019.02.016 PMC8365864

[26] McDermott K Fang M Boulton AJM Selvin E Hicks CW. Etiology, epidemiology, and disparities in the burden of diabetic foot ulcers. Diabetes Care. (2023) 46:209–21. doi: 10.2337/dci22-0043 PMC9797649 36548709

[27] Bury DC Rogers TS Dickman MM. Osteomyelitis: diagnosis and treatment. Am Fam Physician. (2021) 104:395–402. 34652112

[28] Yesil S Akinci B Yener S Bayraktar F Karabay O Havitcioglu H. Predictors of amputation in diabetics with foot ulcer: single center experience in a large Turkish cohort. Hormones (Athens). (2009) 8:286–95. doi: 10.14310/horm.2002.1245 20045802

[29] Lavery LA Ryan EC Ahn J Crisologo PA Oz OK La Fontaine J. The infected diabetic foot: re-evaluating the IDSA diabetic foot infection classification. Clin Infect Dis. (2019) 70:1573–9. 10.1093/cid/ciz489 31179491

[30] Zhang L Long J Jiang W Shi Y He X Zhou Z. Trends in chronic kidney disease in China. N Engl J Med. (2016) 375:905–6. 10.1056/NEJMc1602469 27579659

[31] Akkus G Evran M Gungor D Karakas M Sert M Tetiker T. Tinea pedis and onychomycosis frequency in diabetes mellitus patients and diabetic foot ulcers. A cross sectional - observational study. Pak J Med Sci. (2016) 32:891–5. doi: 10.12669/pjms.324.10027IF:2.2Q3 PMC5017097 27648034

[32] Zhong A Li G Wang D Sun Y Zou X Li B. The risks and external effects of diabetic foot ulcer on diabetic patients: A hospital-based survey in Wuhan area, China. Wound Repair regeneration: Off Publ Wound Healing Soc [and] Eur Tissue Repair Soc. (2017) 25:858–63. 10.1111/wrr.12589 29052949

[33] Sun B Chen Y Man Y Fu Y Lin J Chen Z. Clinical value of neutrophil-to-lymphocyte ratio and prognostic nutritional index on prediction of occurrence and development of diabetic foot-induced sepsis. Front Public Health. (2023) 11:1181880. doi: 10.3389/fpubh.2023.1181880 38026334 PMC10630165

[34] Mariam TG Alemayehu A Tesfaye E Mequannt W Temesgen K Yetwale F. Prevalence of Diabetic Foot Ulcer and Associated Factors among Adult Diabetic Patients Who Attend the Diabetic Follow-Up Clinic at the University of Gondar Referral Hospital, North West Ethiopia, 2016: Institutional-Based Cross-Sectional Study. J Diabetes Res. (2017) 2017:2879249. doi: 10.1155/2017/2879249 28791310 PMC5534295

[35] Saad N Elhadedy K Ramadan N Mohmady O Farid M. The prevalence and risk categorization of diabetic foot complications in cohort group in, Beni Suif, Egypt. Life Sci J. (2013) 3:10.

[36] Yadav R Tiwari P Dhanaraj E. Risk factors and complications of type 2 diabetes. Rev Article. (2008) 9:8–12.

[37] Singh AV Bhardwaj P Upadhyay AK Pagani A Upadhyay J Bhadra J. Navigating regulatory challenges in molecularly tailored nanomedicine. Explor BioMat-X. (2024) 1:124–34.

